# Pediatric Bacterial Meningitis Surveillance in Niger: Increased Importance of *Neisseria meningitidis* Serogroup C, and a Decrease in *Streptococcus pneumoniae* Following 13-Valent Pneumococcal Conjugate Vaccine Introduction

**DOI:** 10.1093/cid/ciz598

**Published:** 2019-09-05

**Authors:** Mamadou Kourna Hama, Dam Khan, Boubou Laouali, Catherine Okoi, Abdoulaye Yam, Moussa Haladou, Archibald Worwui, Peter Sylvanus Ndow, Ricardo Nse Obama, Jason M Mwenda, Joseph Biey, Bernard Ntsama, Brenda A Kwambana-Adams, Martin Antonio

**Affiliations:** 1 Laboratoire Hopital National de Niamey, Niger; 2 World Health Organization (WHO) Collaborating Centre for New Vaccines Surveillance, Medical Research Council Unit The Gambia at the London School of Hygiene and Tropical Medicine, United Kingdom; 3 WHO Country Office, Niamey, Niger; 4 WHO Regional Office for Africa, Brazzaville, Republic of Congo; 5 WHO Intercountry Support Team for West Africa, Ouagadougou, Burkina Faso; 6 Microbiology and Infection Unit, Warwick Medical School, University of Warwick, Coventry, United Kingdom

**Keywords:** meningitis, Niger, cerebrospinal fluid, *N. meningitidis*, *S. pneumoniae*

## Abstract

**Background:**

Meningitis is endemic in Niger. *Haemophilus influenzae* type b (Hib) vaccine and the 13-valent pneumococcal conjugate vaccine (PCV13) were introduced in 2008 and 2014, respectively. Vaccination campaign against *Neisseria meningitidis* serogroup A was carried out in 2010–2011. We evaluated changes in pathogen distribution using data from hospital-based surveillance in Niger from 2010 through 2016.

**Methods:**

Cerebrospinal fluid (CSF) specimens from children <5 years old with suspected meningitis were tested to detect vaccine-preventable bacterial pathogens. Confirmatory identification and serotyping/grouping of *Streptococcus pneumoniae*, *N. meningitidis*, and *H. influenzae* were done. Antimicrobial susceptibility testing and whole genome sequencing were performed on *S. pneumoniae* isolates.

**Results:**

The surveillance included 2580 patients with suspected meningitis, of whom 80.8% (2085/2580) had CSF collected. Bacterial meningitis was confirmed in 273 patients: 48% (131/273) was *N. meningitidis*, 45% (123/273) *S. pneumoniae*, and 7% (19/273) *H. influenzae*. *Streptococcus pneumoniae* meningitis decreased from 34 in 2014, to 16 in 2016. PCV13 serotypes made up 88% (7/8) of *S. pneumoniae* meningitis prevaccination and 20% (5/20) postvaccination. *Neisseria meningitidis* serogroup C (NmC) was responsible for 59% (10/17) of serogrouped *N. meningitidis* meningitis. Hib caused 67% (2/3) of the *H. influenzae* meningitis isolates serotyped. Penicillin resistance was found in 16% (4/25) of *S. pneumoniae* isolates. Sequence type 217 was the most common lineage among *S. pneumoniae* isolates.

**Conclusions:**

*Neisseria meningitidis* and *S. pneumoniae* remain important causes of meningitis in children in Niger. The decline in the numbers of *S. pneumoniae* meningitis post-PCV13 is encouraging and should continue to be monitored. NmC is the predominant serogroup causing *N. meningitidis* meningitis.

## Teaser

Numbers of *Streptococcus pneumoniae* meningitis decreased following 13-valent pneumococcal conjugate vaccine introduction in 2014. The case fatality rate of *S. pneumoniae* meningitis was higher compared with both *Haemophilus influenzae* and *Neisseria meningitidis* meningitis. Increased importance of *N. meningitidis* serogroup C was observed.

Niger is one of the 26 countries situated within the African “meningitis belt,” which spans from Senegal in the west to Ethiopia in the east. Bacterial meningitis is endemic in Niger and the burden of disease is worsened by the periodic occurrence of epidemics in the region, mainly caused by *Neisseria meningitidis*. In 2015, a meningitis outbreak attributed to *N. meningitidis* serogroup C (NmC) occurred, affecting nearly 10 000 people [1–3]. In 2009 and 2006, meningitis outbreaks caused by *N. meningitidis* serogroups A (NmA) and X (NmX), respectively, were reported [4, 5]. *Haemophilus influenzae* and *Streptococcus pneumoniae* are 2 other important pathogens that contribute significantly to the bacterial meningitis burden within Niger [6].

The World Health Organization (WHO) has prioritized the implementation of vaccines that can prevent bacterial meningitis globally, especially those targeting young children. With huge financial support from GAVI, the Vaccine Alliance, the *H. influenzae* type b (Hib) conjugate vaccine and 13-valent pneumococcal conjugate vaccine (PCV13) were introduced into the Niger Expanded Programme on Immunization (EPI) in 2008 and 2014, respectively. Mass vaccination with MenAfriVac, which protects against NmA, was conducted between September 2010 and December 2011 [7]; plans to introduce MenAfriVac into the Niger routine EPI are under way. The coverage for 3 doses of PCV13 in Niger has progressively increased from 13% in 2014 to 76% in 2016 according to WHO/United Nations Children’s Fund estimates, and coverage for the 3 doses of Hib vaccine was estimated to increase from 71% to 80% from 2010 to 2016 [8]. Coverage of the MenAfriVac conjugate vaccine during the vaccination campaign was 76% [7].

Pediatric bacterial meningitis (PBM) surveillance is necessary to monitor the burden and microbiologic etiology of meningitis, particularly within the context of vaccine introduction. The WHO Regional Reference Laboratory (RRL), housed at the Medical Research Council Unit The Gambia at the London School of Hygiene and Tropical Medicine (MRCG), collaborates with WHO to support hospital-based surveillance for invasive bacterial vaccine-preventable disease (IB-VPD) across 10 West and Central African countries, including Niger.

As part of the IB-VPD surveillance network, children <5 years of age with suspected meningitis have cerebrospinal fluid (CSF) specimens collected for culture and latex agglutination at the sentinel-site hospital laboratory. CSF specimens are also sent to the WHO RRL for pathogen detection and serotyping/serogrouping using molecular techniques. The WHO RRL also performs antibiotic susceptibility testing and whole genome sequencing on *S. pneumoniae* isolates to provide insights on antibiotic resistance patterns and molecular epidemiology of *S. pneumoniae*, respectively. We analyzed the surveillance data of IB-VPD from 2010 through 2016 to describe the distribution and the phenotypic and genotypic characteristics of vaccine-preventable bacterial pathogens causing meningitis and to monitor the impact of vaccination.

## MATERIALS AND METHODS

### Surveillance System and Patient Enrollment

The hospital-based surveillance was carried out at the Hopital National de Niamey, a large teaching hospital in Niamey, the capital city of Niger. Patients admitted to the reference hospital come from the 8 different regions in the country. Suspected meningitis cases were defined as sudden onset of fever (>38.5°C rectal or 38.0°C axillary) and combination of any or all of the following clinical symptoms; reduced level of consciousness, stiff neck, and bulging fontanelle if patient is <1 year old. Suspected meningitis cases admitted to the hospital underwent lumbar puncture to obtain CSF for diagnostic testing. Probable meningitis cases wer**e** suspected cases with at least 1 of the following CSF characteristics: turbid appearance, white blood cell count (WBC) of >100 cells/μL or WBC count of 10–100 cells/μL and either an elevated protein (>100 mg/dL) or decreased glucose (<40 mg/dL). Confirmed meningitis cases were those who had a pathogen detected in the CSF collected.

### Laboratory Methods

Based on macroscopic appearance, CSF specimens were classified as clear, turbid, xanthochromic, or blood stained. Microscopic analysis grouped WBC count into 3 groups: ≤10 cells/μL, 10–100 cells/μL, and >100 cells/μL. Gram staining was performed on all CSF specimens collected for the presumptive identification of *S. pneumoniae*, *N. meningitidis*, and *H. influenzae.* Latex agglutination was performed using a Pastorex meningitis kit (Bio-Rad) for detecting Hib, *S. pneumoniae*, and *N. meningitidis* groups A, B, C, Y, and W antigens, following the manufacturer’s instructions. The BINAX NOW kit (Alere), when available, was used for the detection of *S. pneumoniae* antigen. Microbiological culture was done for the isolation of *S. pneumoniae*, *N. meningitidis*, and *H. influenzae.* CSF specimens were streaked on Columbia blood agar and chocolate agar plates for isolation of pure colonies. Antimicrobial susceptibility testing was performed by the disk diffusion method at the sentinel site laboratory, and Etest was done at the WHO RRL. Both methods were done according to the 2017 Clinical and Laboratory Standards Institute guidelines [9].

At the WHO RRL, real-time polymerase chain reaction (qPCR) for autolysin gene (*lytA*), protein D encoding gene (*hpd*), and CU, Zn superoxide dismutase gene (*sodC*) was perfomed for the detection of *S. pneumoniae, H. influenzae*, and *N. meningitidis*, respectively*. Neisseria meningitidis* serogroups A, B, C, W, X, and Y were detected by targeting *sacB*, *synD*, *synE*, *synG*, *xcbB*, and *synF* genes, respectively*. Haemophilus influenzae* serotypes a, b, c, d, e, and f were detected by targeting *acsB*, *bcsB*, *ccsD*, *dcsE*, *ecsH*, and *bexD*, respectively. A total of 37 different *S. pneumoniae* serotypes were targeted using a sequential multiplex qPCR assay [10]. Nontypeable *S. pneumoniae* with cycle threshold values ≤32 by qPCR underwent serotyping by conventional multiplex PCR [11].

### Whole Genome Sequencing Analysis of *S. pneumoniae* Isolates

DNA was extracted from *S. pneumoniae* isolates using a modified QIAGEN kit according to the manufacturer’s instructions. Whole genome sequencing was performed using Illumina Hiseq 2500.

Sequencing reads from each isolate were mapped onto the *S. pneumoniae* ATCC 700669 serotype 23F reference genome using SMALT [12], and pseudo-genomes were placed in a multiple sequence alignment using custom scripts. Single-nucleotide polymorphisms (SNPs) were called from the pseudo-alignment using SNP sites. A maximum likelihood phylogeny was reconstructed with a general time reversible model using randomized accelerated maximum likelihood (RAxML) [13] and visualized in iTOL [14]. Genotypic antimicrobial resistance prediction was also done for the *S. pneumoniae* isolates.

### Statistical Analysis

Patient data were entered in an Epi Info database tool at the sentinel site and sent to the WHO RRL where PCR data were entered. Fisher exact test was done using Stata version 12 software (StataCorp, College Station, Texas) to determine associations between CSF characteristics and PCR results. Percentages were calculated in Microsoft Excel software and presented on tables and as prose.

### Ethical Considerations

Ethical approval was not a requirement in Niger for routine meningitis surveillance, including drug susceptibility testing of collected isolates, as this was approved within the routine diagnostic algorithm at the Ministry of Health. However, informed consent was sought from the parents or guardians of the surveillance participants. Additionally, the surveillance received overarching ethical approval (SCC1188) by the joint Medical Research Council/The Gambia Government ethics board that allowed the analysis of collected West African isolates at MRCG.

## RESULTS

A total of 2580 suspected meningitis cases among children <5 years of age were reported during the 2010–2016 surveillance period (Table 1). More than half of the cases were males and more than a third of children were in their first year of life. We screened 81% (2085/2580) of suspected cases for bacterial pathogens at the sentinel site and/or at the WHO RRL. Of this number, 13% (273/2085) were confirmed meningitis cases; 48% (131/273) *N. meningitidis*, 45% (123/273) *S. pneumoniae*, and 7% (19/273) *H. influenzae*. None of the infants that had confirmed *S. pneumoniae* had a record of PCV13 vaccination. However, it is important to note that the PCV13 vaccination record is unknown for 78.2% (2017/2580) of participants in the surveillance.

**Table 1.** Characteristics of Children Aged <5 Years Admitted at Hopital National de Niamey With Suspected Meningitis (N = 2580)

Characteristic	No.	(%)
Age, mo		
0–11	1106	(42.9)
12–23	431	(16.7)
24–59	1006	(39.0)
Unknown	37	(1.4)
Sex		
Male	1539	(59.7)
Female	1017	(39.4)
Unknown	24	(0.9)
Antibiotic before admission		
Yes	127	(4.9)
No	375	(14.5)
Unknown	2078	(80.5)
Final outcome		
Discharged alive	1349	(52.3)
Died	263	(10.2)
Unknown	968	(37.5)

In total, 339 CSF samples were sent to the WHO RRL for serotyping/serogrouping during the surveillance period (Table 2). Prior to PCV13 introduction, the WHO RRL only received 2% of CSF specimens from suspected cases, whereas after PCV13, 22% of CSF specimens were sent to WHO RRL for PCR. *Streptococcus pneumoniae* meningitis decreased from 34 cases in 2014 to 16 cases in 2016. Overall, 88% (7/8) of all *S. pneumoniae* meningitis cases prior to PCV13 (2010–2013) that were serotyped were caused by PCV13 serotypes. PCV13 serotypes were only responsible for 20% (5/25) of *S. pneumoniae* serotyped cases post-PCV13 (2014–2016). Serogroup results for 17 *N. meningitidis* cases were obtained. NmC was responsible for 59% (10/17) and *N. meningitidis* serogroup W was responsible for 35% (6/17). The NmC cases were all detected in 2016. No NmA cases were reported throughout the surveillance. *Haemophilus influenzae* meningitis was uncommon, with 19 cases reported. Out of this number, 84% (16/19) were <1 year old. Of the 3 confirmed cases with serotype data, 2 were Hib.

**Table 2.** Cerebrospinal Fluid Specimens Received and Tested at the World Health Organization Regional Reference Laboratory

Year	Total WHO RRL Cases, No.	CSF Samples Received/Tested at WHO RRL, No.	No. of Confirmed Cases, No.	*Haemophilus influenzae*, No. (%)	*Streptococcus pneumoniae*, No. (%)	*Neisseria meningitidis*, No. (%)
2010	459	6	1	0 (0)	1 (100)	0 (0)
2011	210	6	5	1 (20)	4 (80)	0 (0)
2012	214	4	3	1 (33)	2 (67)	0 (0)
2013	223	1	0	0 (0)	0 (0)	0 (0)
2014	488	124	30	0 (0)	24 (80)	6 (20)
2015	584	97	6	1 (17)	3 (50)	2 (33)
2016	402	101	20	0 (0)	9 (45)	11 (55)
Total	2580	339	65	3 (5)	43 (66)	19 (29)

Abbreviations: CSF, cerebrospinal fluid; RRL, Regional Reference Laboratory; WHO, World Health Organization.

**Figure 1.**
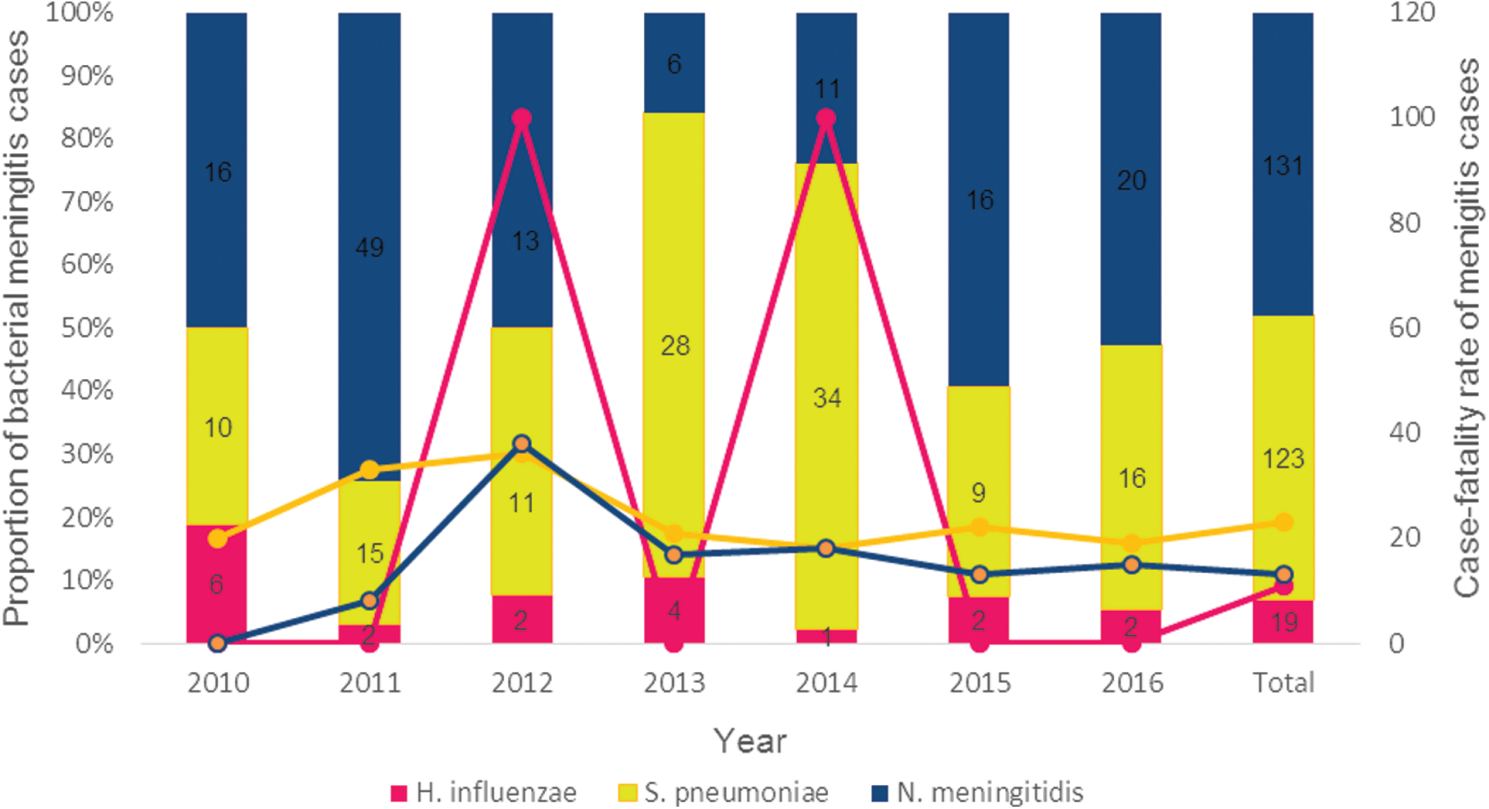
Bar graph showing yearly distribution of *Streptococcus pneumoniae*, *Neisseria meningitidis*, and *Haemophilus influenzae.* The line graph on the secondary axis shows the case fatality rate of the 3 pathogens.

**Figure 2.**
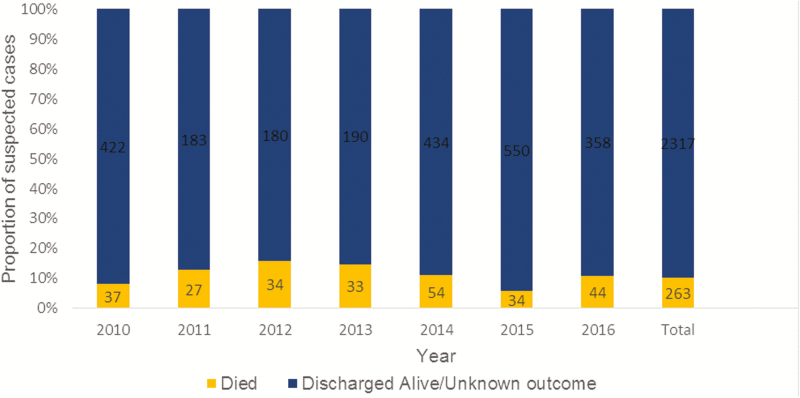
Outcome of meningitis cases by year.

Disaggregating the results by year, *N. meningitidis* dominated except in 2013 and 2014 when *S. pneumoniae* was the most common pathogen (Figure 1). When the data were segregated by month, the highest number of suspected meningitis cases was reported during March and April, with 26% (681/2580) of suspected cases occurring in these 2 months. *Streptococcus pneumoniae* was the deadliest pathogen, with a case fatality rate (CFR) of 23%. The CFRs of *N. meningitidis* and *H. influenzae* were 13% and 11%, respectively. The number of suspected meningitis cases admitted and the total number of children who died fluctuated over the years of the surveillance (Figure 2). The CFR was highest in 2012 (16%).

Among confirmed meningitis cases with recorded data, 84% (126/150) had seizures, 65% (70/107) had reduced level of consciousness, and 69% (77/111) had neck stiffness. Among confirmed cases, 33% (19/57) presented with bulging fontanel, and 90.0% (17/19) of this number were <2 years old.

 The majority of patients (67% [1588/2372]) had a clear CSF and <1% (12/2372) had a xanthochromic CSF specimen. A strong association between turbidity of CSF and a positive PCR result was noted (*P* < .001). For instance, a pathogen was detected in 71% (40/56) of the turbid CSF specimens tested. Likewise, patients with a WBC count of >100 cells/μL were more likely to be infected with a bacterial pathogen (*P* < .001) compared with patients with WBC count <100 cells/μL.

**Figure 3.**
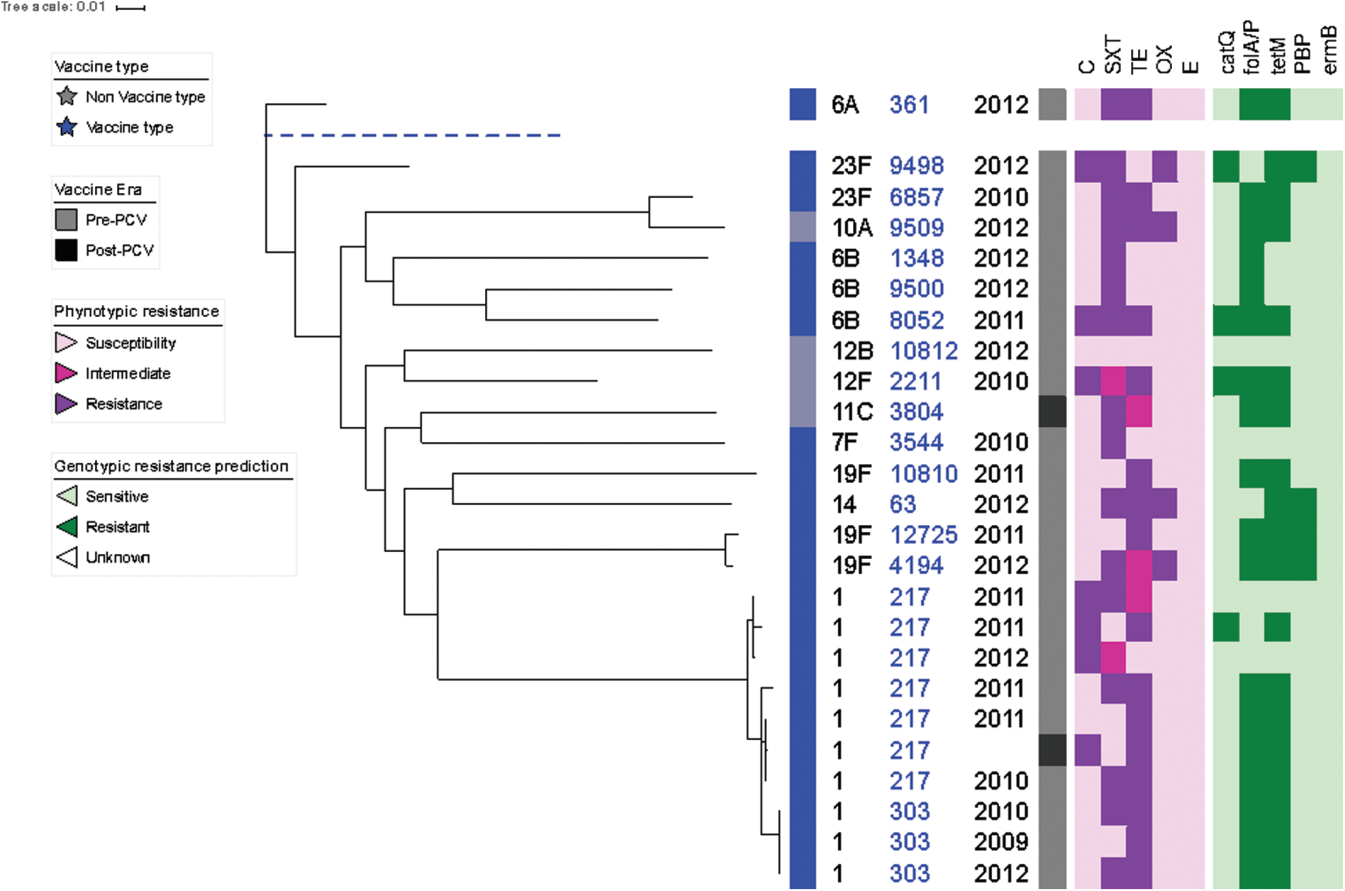
Maximum likelihood whole genome phylogenetic tree of *Streptococcus pneumoniae* isolates recovered from cerebrospinal fluid. Tree annotation from left to right: vaccine type; serotype; multilocus sequence type; year of admission; vaccine era (pneumococcal conjugate vaccine); phenotypic antibiotic resistance profiles to chloramphenicol, trimethoprim-sulfamethoxazole (SXT), tetracycline, oxacillin, and erythromycin; and the presence/absence of the antibiotic resistance genes *catQ* (chloramphenicol), *folA/ folP* (SXT), *tetM* (tetracycline), *PBP* (penicillin), and *ermB* (erythromycin). Abbreviations: C, chloramphenicol; E, erythromycin; OX, oxacillin; PCV, pneumococcal conjugate vaccine; SXT, trimethoprim-sulfamethoxazole; TE, tetracycline.

Phylogenetic analysis and antimicrobial susceptibility testing was performed for 25 *S. pneumoniae* isolates (Figure 3). Of the 25 *S. pneumoniae* isolates, 23 were collected in the pre-PCV13 era and 2 were collected in the post-PCV13 era. PCV13 *S. pneumoniae* made up 84% (21/25) of all the isolates collected. Serotype 1 was the most frequently detected *S. pneumoniae* serotype among the isolates (40% [10/25]). The data also show a close relationship among serotype 1 isolates, all of which belonged to clonal complex 217 (CC217). Resistance to trimethoprim-sulfamethoxazole (SXT) and tetracycline was widespread, with 72% (18/25) of *S. pneumoniae* isolates expressing resistance to SXT and 76% (19/25) expressing resistance to tetracycline. Resistance to tetracycline was common among serotype 1 isolates, with 80% (8/10) expressing resistance. We found that 16% (4/25) of *S. pneumoniae* isolates were resistant to penicillin. In total, there were 6 resistance genotypes that were detected by genotypic resistance prediction and not expressed in their corresponding phenotypic resistance profiles. The *folA/P* gene (mutations within the gene confers resistance against SXT) was the gene detected for 4 such cases.

## Discussion

*Neisseria meningitidis* was the predominant etiologic agent among confirmed meningitis cases, although there were some years that *S. pneumoniae* meningitis exceeded the number of *N. meningitidis* meningitis. *Streptococcus pneumoniae* was the deadliest pathogen with CFR of 23%. We found some resistance to penicillin, and high resistance to SXT was observed among *S. pneumoniae* isolates.

We have reported data from hospital-based surveillance describing the magnitude and etiology of suspected meningitis among children <5 years old admitted to a large tertiary care hospital in Niger from 2010 to 2016. Surveillance data are helpful to inform and evaluate immunization policies by describing the epidemiology of the disease in the country and monitor impact.

Real-time PCR is more sensitive than culture-based methods and was thus used at the WHO RRL for confirming cases as well as providing serotyping data [15]. Comprehensive serotyping data are crucial for evaluating the impact of the vaccines targeting bacterial meningitis pathogens. The overall detection of the 3 pathogens appears to have decreased following mass vaccinations with MenAfriVac and introduction of Hib and PCV13 in the infant immunization program in Niger. A 2002–2008 surveillance carried out by Maïnassara and colleagues found *S. pneumoniae*, *N. meningitidis*, and *H. influenzae* detection rate of 40.81% (5229/12811) [6], whereas we present a detection rate of 13.1% (273/2085).

Before the mass immunization campaign against NmA, *N. meningitidis* was by far the most common cause of meningitis in Niger [16, 17]. However, our data show the increasing importance of *S. pneumoniae* as a causative agent of meningitis in Niger. It was almost as prevalent as *N. meningitidis*. Furthermore, in 2013 and 2014, there were more *S. pneumoniae* meningitis than *N. meningitidis* cases. The decrease in *N. meningitidis* meningitis can be attributed to the effectiveness of the MenAfriVac vaccine. However, the serogroup A specificity of MenAfriVac has raised concerns that other serogroups may arise as an important cause of *N. meningitidis* meningitis in the African meningitis belt regions. Based on our analysis, NmC now represents >50% of *N. meningitidis* identified. This is a concern given that NmC has the potential to cause large outbreaks as demonstrated in Nigeria with the emergence of a previously unknown hypervirulent NmC strain [18]. The same strain was the etiologic agent of the meningitis outbreak in Niger in 2015. A monovalent conjugate vaccine against NmC is currently licensed for use. Vaccination is the most effective strategy for reducing incidence of NmC meningitis as demonstrated in countries with routine use of monovalent C conjugate vaccine [19]. However, due to cost consideration in African countries, the NmC vaccine is only rolled out during outbreaks, targeting the most affected age groups and districts. At the moment, the best approach to control outbreaks caused by this strain in Niger is to strengthen surveillance and put response systems in place. This would ensure timely diagnosis and management of case contacts to minimize the risk of transmission.

Our data also show the decline in *H*. *influenzae* meningitis when compared to the pre-Hib vaccine era [20]. However, 19 cases were still detected in the surveillance and the most vulnerable population was children <1 year old. Attempts to further improve vaccine coverage to further reduce this number should be a priority. Countries such as Senegal and The Gambia have reported a near elimination of Hib meningitis [21, 22].

We have shown that the number of confirmed *S. pneumoniae* meningitis cases decreased after 2014. However, due to the small number of *S. pneumoniae* meningitis cases with serotype information available pre-PCV13, we were unable to compare serotype distribution pre- and post-PCV13. More data are needed to show changes in serotype distribution. The high CFR of *S. pneumoniae* meningitis is in accordance with studies done in the country prior to PCV13 introduction [17]. Although cases of *S. pneumoniae* meningitis decreased after 2014, this was not followed by a concurrent decrease in the CFR of *S. pneumoniae*. The CFR remained fairly stable throughout the surveillance period. Sequence type 217 clonal complex, common in Africa [23, 24], was the most dominant *S. pneumoniae* lineage in our surveillance. The lineage was the etiologic agent of the 2005 meningitis outbreak in the Kassena-Nankana district within Ghana [25].

Infections caused by *S. pneumoniae* resistant to penicillin have increased at an alarming rate over the years [26–28]; this trend was also noticed in our surveillance, as we detected 4 isolates that were resistant to oxacillin (a penicillin-class antibiotic). Penicillin resistance of carriage *S. pneumoniae* isolates was also observed among children <2 years old in Niger [29]. We found high resistance of *S. pneumoniae* isolates to SXT, which could be explained by their indiscriminate and widespread use in Africa. A study conducted in Finland has shown that the development of *S. pneumoniae* resistance to SXT is affected by the consumption rate of the antibiotic [30]. SXT is not used to treat meningitis in Niger. However, the Joint United Nations Programme on HIV/AIDS and the World Health Organization recommended the antibiotic to be used for prophylaxis in both children and adult living with human immunodeficiency virus (HIV)/AIDS in Africa to prevent severe bacterial infections, opportunistic infections, and malaria [31]. Strategies such as sensitization programs should be prioritized to reduce the indiscriminate consumption of SXT among persons living without HIV or those without severe or advanced HIV clinical disease.

A major limitation of this analysis is the low number of CSF specimens sent to the WHO RRL for analysis prior to PCV13 introduction. This limited our ability to evaluate changes in serotyping distribution post-PCV13. In addition, the surveillance is hospital-based and our observations cannot be generalized to the rest of the country. Last, vaccination history was not recorded for the majority of cases.

## Conclusions

We have shown a reduction in *S. pneumonia*e meningitis following PCV13 introduction. However, we were unable to properly evaluate changes in serotype distribution of *S. pneumoniae* meningitis because not all cases of *S. pneumoniae* meningitis were serotyped. We have also shown the emergence of NmC as an important cause of meningitis. The findings of this analysis support the need to improve and continue surveillance of meningitis and characterization of bacterial pathogens to provide additional data for further analysis to better understand the epidemiology of meningitis in Niger.

## Notes

***IBD Writing Group members.*** Brenda Kwambana-Adams, Madikay Senghore, Effua Usuf, Archibald Worwui, Egere Uzochukwu, Akram Zaman, Catherine Okoi, Florian Gehre, Leopold Tientcheu, Nuredin Ibrahim Mohammed, Felix Dube, Peter Ndow, Jason M. Mwenda, Suso Sambou, Sheikh Jarju, Dam Khan, Ebruke Chinelo, Rowan Bancroft, and Martin Antonio.

***Author contributions.*** M. A. and J. M. M. established the WHO Regional Office for Africa–supported Paediatric Bacterial Meningitis Surveillance Network in West Africa. M. A. supervised the overall network including setting up the sentinel surveillance system. M. H. H. and B. L. clinically investigated and recruited the patients at the sentinel sites, collected demographic data, performed microbiological testing at sentinel sites, and shipped cerebrospinal fluid and bacterial isolates to WHO RRL at MRCG for confirmatory testing and molecular analysis (supervised by B. A. K.-A. and M. A.). B. A. K.-A. and M. A. developed the analysis plan and contributed to analysis and interpretation of data along with the IBD Writing Group. D. K., B. A. K.-A., and M. A. drafted the manuscript along with M. A. S. All authors contributed to the interpretation of the findings and the writing of the final manuscript.

***Acknowledgments.*** The authors thank the WCO of Niger and the World Health Organization (WHO) Intercountry Support Team for coordination, advice, and support throughout the project; the surveillance participants and their families in Niger; and the staff members and students at the WHO Regional Reference Laboratory (RRL), Medical Research Council Unit The Gambia (MRCG) at the London School of Hygiene and Tropical Medicine, as well as the Invasive Bacterial Diseases (IBD) Writing Group, for their advice and input. The data sets supporting the conclusions of this article are included within the article.

***Financial support.*** Financial support for sentinel site surveillance was provided by the Niger Ministry of Health, GAVI - the Vaccine Alliance, through a grant to the WHO for the African Paediatric Bacterial Meningitis Surveillance Network.

***Supplement sponsorship.* This supplement was supported with funds from Gavi, the Vaccine Alliance through The World Health Organization and the CDC Foundation, and The Medical Research Council Unit The Gambia at the London School of Hygiene and Tropical Medicine.**

***Potential conflicts of interest.*** The authors report no potential conflicts of interest. All authors have submitted the ICMJE Form for Disclosure of Potential Conflicts of Interest. Conflicts that the editors consider relevant to the content of the manuscript have been disclosed.
